# Microstructural Investigations of Ni-Based Superalloys by Directional Solidification Quenching Technique

**DOI:** 10.3390/ma13194265

**Published:** 2020-09-24

**Authors:** Tobias Wittenzellner, Shieren Sumarli, Helge Schaar, Fu Wang, Dexin Ma, Andreas Bührig-Polaczek

**Affiliations:** 1 Foundry Institute, RWTH Aachen University, 52072 Aachen, Germany; shieren.sumarli@rwth-aachen.de (S.S.); h.schaar@gi.rwth-aachen.de (H.S.); D.Ma@gi.rwth-aachen.de (D.M.); sekretariat@gi.rwth-aachen.de (A.B.-P.); 2 State Key Laboratory for Manufacturing System Engineering, School of Mechanical Engineering, Xi’an Jiaotong University, Xi’an 710049, China; fuwang@xjtu.edu.cn

**Keywords:** nickel-based superalloy, microstructure, phase transformation, directional solidification, quenching

## Abstract

The improvement of the mechanical properties of Ni-based superalloys is achieved in most cases by modifying the chemical composition. Besides that, the processing can be modified to optimize the as-cast microstructure with regard to the mechanical properties. In this context, the present study highlights the solidification mechanism of several Ni-based superalloys by conducting experiments using a modified, laboratory-scale Bridgman-Stockbarger furnace. In that context, the single-crystal rods are partially melted, directionally solidified and quenched sequentially. Several characterization methods are applied to further analyze the influence of the alloying elements and the variation of the withdrawal rate on the as-cast microstructure. Four stages of solidification are distinguished whereby the morphology observed in the different stages mainly depends on the cooling rate and the local concentration of the carbide forming elements. The effect of carbide precipitation and the effect on the as-cast microstructure is investigated by employing energy dispersive X-ray spectrometry (EDX) and electron backscatter diffraction (EBSD) analysis techniques. A local polycrystalline structure is observed in the single-crystal system as consequence of the influence of the carbon content and the cooling rate. The present work aims to develop strategies to suppress the formation of the polycrystalline structure to maintain the single-crystal microstructure.

## 1. Introduction

Ni-based superalloys, based on the superior thermo-mechanical material properties, are widely used in technical high temperature applications like aviation, chemical industry or energy generation industry. Since the requirement of materials with exceptional mechanical properties increases, similarly the development of materials which can withstand these harsh conditions is advanced. The earliest development began with changing the structure of Ni-based superalloys from polycrystalline to columnar and then single crystal. The intention is to completely eliminate grain boundaries so that failure by grain boundary sliding as well as excessive diffusion and the resulting segregation are avoided. In addition, chemical composition alteration by adding, removing or replacing several alloying elements such as carbon and refractory metals are carried out as well [1,2,3,4]. This development resulted in six different generations of Ni-based single-crystal superalloys whereby the first and second generation are most used in industrial application [5,6,7,8,9]. 

By optimizing the solidification process, the performance of the Ni-based superalloys can be further improved. The solidification process is complex due to the multicomponent system with up to 14 alloying elements [10]. Therefore, in these systems, strong segregation effects can be observed that lead to the formation of eutectic structures, which is detrimental in respect to the mechanical properties. As consequence, heat treatment processes are applied to homogenize the heterogeneous as-cast microstructure [11]. In spite of that, some phases are indissoluble during heat treatment and, therefore, it is favorable to produce an optimum structure from the beginning. To control the solidification process, a thorough investigation of the solidification behavior of the system is essential to combine theoretical and practical approaches. Prior studies of the solidification behavior can be traced back to the last decades. Various methods have been established and employed to analyze the solidification behavior of superalloys such as Differential Scanning Calorimetry (DSC), analytical modelling and numerical simulation as well as in situ observation [11,12,13,14,15,16,17,18].

Several studies investigated the solidification process under equilibrium conditions for various Ni-based superalloys using the Calphad method [15,17]. This method is, however, unable to predict phase transformations under non-equilibrium condition as in the case of real solidification, for example the formation of the *γ*/*γ*’ eutectic. Other prior investigations involved three-dimensional (3-D) reconstructions from a series of two-dimensional (2-D) planar transverse sections to define the phase formation sequence in Ni-based superalloys [14,16]. Besides the extent of this method being restricted to one particular phase, it is also fairly complicated to be perceived dynamically. Other studies investigated the solidification behavior of superalloys using DSC to determine the enthalpy and heat capacity by heating and cooling the system [12,18]. However, when test substrates produce thermal effects, the cooling or heating rate becomes nonlinear and, hence, the coefficient of correlation is hardly deduced and implemented in quantitative calculation. On top of that, sensitivity and accuracy may be reduced by thermal exchange among the test substrate, reference material and environment [11].

Nowadays, numerical approaches such as the phase field modelling are widely accepted based on various mathematical models to simulate and analyze solidification behavior under settled critical conditions [13]. The simulations inherit a series of assumptions and logical postulations that can limit the predictive power of the obtained results. Even though there are many available methods to study the solidification behavior of superalloys, they possess one major and common shortcoming: incapacity of real-time or timestamped structure observation. This drawback could be overcome by the use of in situ study employing Synchrotron X-ray Diffraction; in that regard, the configuration is relatively complicated. To circumvent the complexity of in situ studies, several scientific works reported the quenching of the material to freeze the mushy zone formed throughout the directional solidification [11,19]. Based on that, the corresponding solidification morphologies can be closer investigated using available characterization techniques. 

Considering the abovementioned evidences, the quenching method is implemented in the present study. Further, an additional thermocouple wire is mounted in each test substance so that the identification of the phase formation temperatures becomes more precise. Based on various deficiencies of the mostly applied analyzing techniques, the formation sequence of multiple phases during solidification of Ni-based superalloys has not yet been sufficiently explored. In the present paper, much attention is drawn to precisely reveal the real-time microstructure evolution of several Ni-based superalloys, more specifically for CMSX-6, CMSX-6+0.03C and MAR-M247. Since the processing has a strong influence on the solidification process, the effect of withdrawing process on the final microstructure is closer investigated by applying systematically varied withdrawal velocities. Furthermore, the focus of the present work is on the evaluation of the effect of carbon addition on the microstructure and the resulting material properties of the superalloys. As reported in literature for the MAR247 alloy, the formation of carbides is beneficial in view of the high-temperature material properties since grain boundary sliding is impeded by pinning effects [20,21]. It can be assumed that this alloying approach can be transferred to other commercial superalloys like CMSX-6. A lot of investigations on the microstructure of this alloy have already been done, but no addition of carbon was researched on CMSX-6 [22,23,24]. In that context, prior investigations solely considered carbides as an individual phase, disregarding the possible influence on the formation of other phases. To account for this correlation of the carbide precipitation and the *γ* matrix as well as the *γ*/*γ*’ eutectic is one of the main objectives of the present study. 

## 2. Materials and Methods

**Figure 1:**
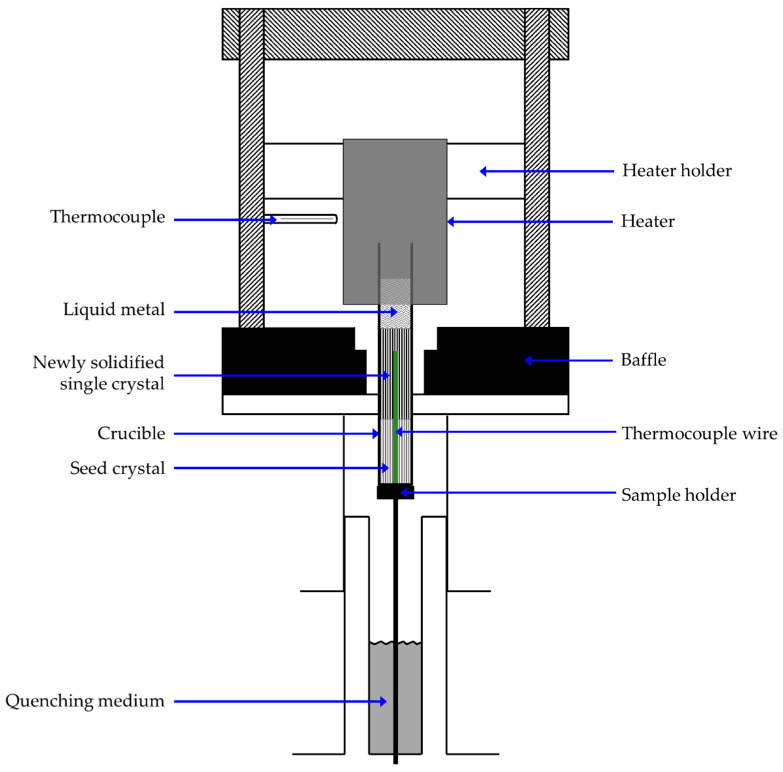
Schematic of the modified, laboratory-scale Bridgman-Stockbarger furnace.

The materials investigated in this study were CMSX-6, CMSX-6+0.03C and MAR-M247, whose compositions are listed in Table 1. CMSX-6 is a carbon free Ni-based superalloy, but due to the production of the single crystals a small amount of 0.006 wt.% of carbon is contained. To investigate the direct influence of carbon on the microstructure, 0.03 wt.% of carbon was added to CMSX-6 alloy (CMSX-6+0.03C). The third alloy used was the commercial MAR-M247, which has an even higher carbon content. These were in the form of single-crystal rods, which had been pre-cast in a conventional Bridgman furnace, with a diameter of 15 mm and a height between 95–105 mm. A 60 mm hole was eroded into each single crystal to install a B-type thermocouple and the samples were placed in a cylindrical alumina crucible. The experiments were performed in a laboratory-scale furnace adopting the Bridgman-Stockbarger furnace, schematically shown in Figure 1. The technique enabled the furnace to consecutively partially remelt and directionally solidify the material. The unmelted part acted as a seed crystal for the subsequent directional solidification (DS). Some adjustments were made so that throughout the process the crucible containing raw material was in a stationary condition whilst the thermal field, i.e., the furnace, could be in motion vertically as needed. Moreover, the crucible did not rotate during withdrawal and the furnace design was altered allowing quenching to be performed right after withdrawal was completed.

Before the procedure began, the furnace was flushed with high-purity argon gas for 80 min to prevent oxidation of the graphite heater and holder, as well as to protect the liquid metal from contamination. During the experiment, the charge was placed on a sample holder and the furnace, which had been preheated to 1773 K (1500 °C), was lowered such that the part of the charge to be melted was encircled by the graphite heater. The furnace was held in this position for 10 min to ascertain uniform melting and homogenize the melt. The furnace was shifted up 50 mm with two alternatives of velocity, hereinafter referred to as withdrawal rate *V*, i.e., 1 and 4 $\mathrm{mm}\cdot \min^{-1}$ (W1 and W4). After the desired position was reached, the charge was rapidly quenched in a liquid metal of Ga–In system closest to eutectic, with 24.6 wt.% or 16.5 at.-% of In. From the recorded temperature data, thermal gradients *G* of 8 $K\cdot {\mathrm{mm}}^{-1}$ (for 1 $\mathrm{mm}\cdot \min^{-1}$) and 6 $K\cdot {\mathrm{mm}}^{-1}$ (for 4 $\mathrm{mm}\cdot \min^{-1}$) at the solidification front were evaluated. As cooling rate $\dot{T}$ is the product of *V* and *G*, the corresponding cooling rates of 0.13 $K\cdot s^{-1}$ and 0.4 $K\cdot s^{-1}$ at the liquid-solid interface (L/S) were calculated.

Based on preliminary analysis, which unveiled the exact portion of mushy zone and single crystal regions, the rods were sectioned transversally (perpendicular to the growth direction) and longitudinally (parallel to the growth direction). Four samples were generated from each rod, indicating transversal cuts of mushy zone and single crystal regions (TC-MZ and TC-SC) as well as longitudinal cuts of these regions (LC-MZ and LC-SC). The samples were subsequently hot-embedded and metallographically prepared for further analyses. Additionally, a vibratory polisher delivering a scratch-free surface was utilized right before metallographic examinations. If necessary, waterless Kalling’s reagent containing 80 mL of ethanol, 40 mL of HCl and 2 g of Copper (II)Chloride was used as the etching solution to accentuate the structure and intended phases. During observation of each LC-MZ sample under Zeiss Axio optical microscope (OM, Carl Zeiss Microscopy GmbH, Oberkochen, Germany) at 200× magnification, the earliest visible forms of phases were marked to indicate the phase transformation points and later calculate the formation temperatures.

Some images of unetched samples were taken, in which the amount of porosity and carbide fraction were analyzed quantitatively employing an image processing software ImageJ (NIH, Bethesda, MD, USA). The microstructure development of the *γ*/*γ*’ eutectic island and the precipitated *γ*’ phase was captured using Zeiss Ultra-55 scanning electron microscope (SEM, Carl Zeiss Microscopy GmbH) in Aachen/Germany. ImageJ was also utilized to support the measurement of volume fraction of *γ*’ precipitates. Chemical characterization of the formed carbides was determined by energy dispersive X-ray spectrometry (EDX) analysis technique in either Zeiss Ultra-55 SEM or Zeiss 1540 XB Crossbeam SEM (Carl Zeiss Microscopy GmbH) in Aachen/Germany. The crystallographic orientation relationship between *γ*/*γ*’ eutectic and other structures, i.e., *γ* matrix and carbides, in LC-SC samples of CMSX-6+0.03C were examined by electron backscatter diffraction (EBSD) technique using Zeiss 1540 XB Crossbeam SEM.

## 3. Results and Discussion

### 3.1. Solidification Sequence of the Formed Phases

**Figure 2:**
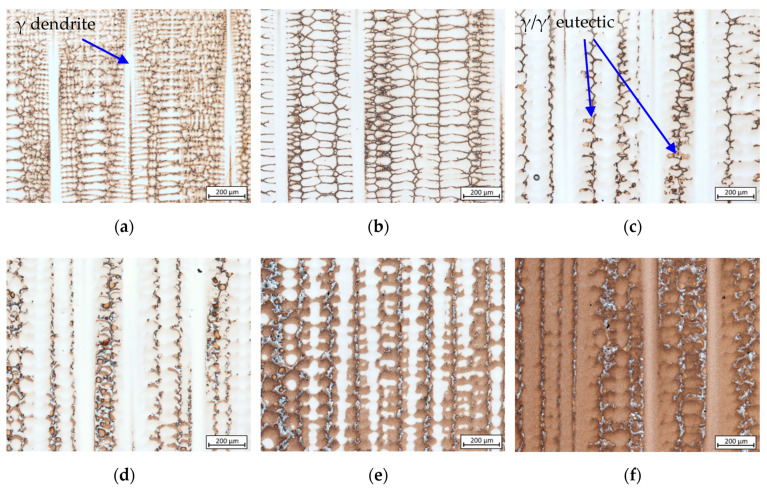
Microstructure evolution observed during preliminary analysis of CMSX-6 (W4) in sequence the (**a**–**e**) mushy zone from dendrite tip to (**f**) solidified single crystal.

**Figure 3:**
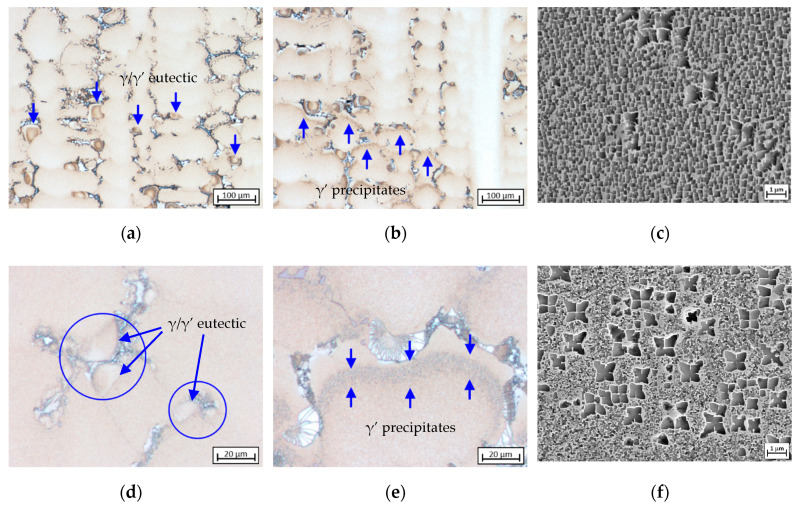
Microstructure images indicating: (**a**,**d**) *γ*/*γ*’ eutectic formation point; (**b**,**c**,**e**,**f**) the earliest precipitation of *γ*’ phase. Images (**a**,**b**,**d**,**e**) represent the LC-MZ sample of CMSX-6+0.03C (W4), (**c**) the CMSX-6+0,03C (W1) and (**f**) MAR-M247 (W1), respectively.

All six rods possess similar-looking mushy zones with a typical dendrite array, i.e., dendrites growing concurrently in one specific direction. Figure 2 depicts an example of the analysis result of CMSX-6 (W4) indicating that along the height of mushy zone, some phases emerged gradually one after another. The formed phases are increasing in size and developing in shape along with the formation of another phase. There are four prevalent phases emerged along the mushy zone, namely *γ* matrix, carbides, *γ*/*γ*’ eutectic and *γ*’ precipitates. The solidification sequence is elaborated as follows:

1. Crystallization of *γ* matrix: $L_{1}\to L_{2}+\gamma$ at 1476.88–1521.15 K characterized by dendrite tips as seen in Figure 2a. According to the liquidus temperatures of 1610 K (CMSX-6 and CMSX-6+0.03C) and 1652 K (MAR-M247), the existence of undercooling by 103.27–134.26 K is evident since crystallization was initiated bellow the supposed temperatures.
2. Solidification of fine equiaxed carbides in the interdendritic area: $L_{2}\to L_{3}+\gamma +MC$ at 1442.58–1496.56 K.
3. Formation of *γ*/*γ*’ eutectic: $L_{3}\to \gamma /\gamma^{\prime }$ and $L_{3}+\gamma \to \gamma /\gamma^{\prime }$ at 1418.26–1472.56 K as depicted in Figure 3a,d.
4. Solid-state phase transformation: $\gamma \to \gamma^{\prime }$ at 1405.68–1470.29 K. The precipitation of *γ*’ phase is indicated by dark-brown patches formed around the dendrite lobes as shown in Figure 3b,e, which was confirmed by SEM analysis results (Figure 3c,f) and is consistent with previous findings [11,25].

The lower crystallization temperature of *γ* matrix compared to prior studies might be caused primarily by the different analysis methods used such as DSC and Thermo-Calc calculation [12,15,18,26]. Since this study involved real-time structure observation that is able to disclose the existence of undercooling in non-equilibrium systems, the shortfalls owned by other methods are eliminated. Small needle-like or script-shaped carbides (<5 μm) were found in the quenched liquid above the dendrite tips in all samples. However, equiaxed carbides as sign of homogeneous nucleation were not observed there as it was reported previously by Chen et al. [20]. On that account, these carbides are evidently considered as the product of quenching process. On the other hand, some fine equiaxed carbides were present in the interdendritic region a few mm below the dendrite tips, indicating that the carbides grew directly from the melt, which is in agreement with previous studies [19,27,28]. These carbides are, however, extremely difficult to discover, and thus the appearance of small blocky carbides spotted in the dendrite arms slightly further down the mushy zone, particularly in the cortex region, were specified as the formation point instead of those in the interdendritic region.

Since there were not any carbides in the dendrite core, the carbides observed around the dendrite arms nucleate in the interdendritic region [29]. As *γ* dendrites continuously grew such that the interdendritic region was consumed by the growing dendritic region, at some point the dendrites would come into contact with the already nucleated carbides in the interdendritic region. Keeping on growing transversely, the dendrites encompassed most of the carbides and the remaining carbides were only partially enveloped [25]. Observations found that the size of interdendritic region right above the formation point of *γ*/*γ*’ eutectic was small compared to that of the formed eutectic islands. Thus, the eutectic formation could not have involved eutectic reaction, since an adequate amount of liquid melt as well as space for the corresponding size of eutectic islands to nucleate and grow were not provided by the interdendritic region. Prior studies confirm that the eutectic formation includes a combination of eutectic and peritectic reactions, through which *γ*’ nucleates from primary *γ* phase at *γ*-liquid interface as their lattice misfit is small and it later on grows into both remaining liquid and *γ* dendrite [12,26,30].

### 3.2. Shrinkage Porosity

**Figure 4:**
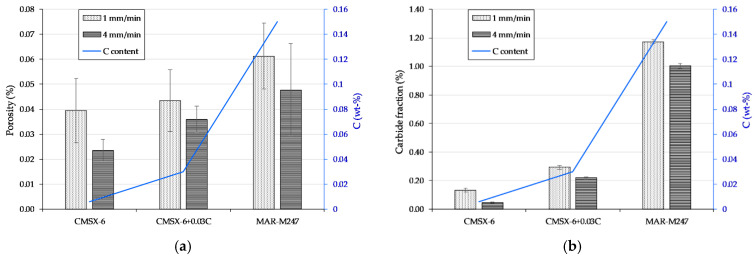
Dependence of carbon content on the (**a**) shrinkage porosity and (**b**) carbide fraction of single crystal part of the samples directionally solidified at different rates.

Since voids contained within the samples were irregular in both shape and size, their formation was obviously a consequence of solidification shrinkage. In Figure 4a, porosity results for single crystal parts directionally solidified at both rates are presented. It indicates that porosity is strongly influenced by carbon content in the alloy and therefore the amount of carbide formed throughout the structure. Increasing carbon content leads to an increase in porosity, with an average value of 0.061% and 0.024% being the highest and lowest found in MAR-M247 (W1) and CMSX-6 (W4), respectively. However, the existence of porosity in samples with higher cooling rate was slightly dampened by roughly 0.008–0.016%. This reduction in porosity is associated with a decrease in carbide fraction at higher cooling rate. During DS, shrinkage occurs around the solidifying crystal while the remaining liquid above gravitationally flows to compensate the shrinkage. The presence of carbides in the interdendritic region significantly reduces its permeability, which leads to an elevated amount of porosity with increasing carbon content and thus carbide fraction [27,29].

Contradictory findings revealed that an addition of carbide decreased porosity, which was proven by pores enclosed by carbides or suggested that carbides precipitate in the solid instead of the liquid [28,31]. Since carbides have a larger cell size than *γ* and *γ*’, the microstructural expansion was proposed to decrease the amount of resulting shrinkage porosity. However, it was rather unjustifiable if pores were entirely enveloped by carbides, which would indicate that carbides were nucleated on the pores or they were trapped in the carbides. Carbides are commonly precipitated at temperatures close to the liquidus, while pores are formed at end of solidification where filling by the remaining liquid is not possible. The alongside existence of carbides and pores could be caused by carbides blocking the fluid flow, on the other side of which shrinkage occurs and therefore pores are built up. Partial or complete envelopment of carbides could also indicate that the cores consist of foreign substances such as oxides instead of pores [19,32]. 

### 3.3. Carbide Solidification at Different Compositions and Rates

#### 3.3.1. Fraction

Carbide fraction measurement was carried out on the single crystal part of all samples, with results presented in Figure 4b. An increase from 0.13% to 0.29% to 1.17% and 0.05% to 0.22% to 1.00% for the CMSX-6, CMSX-6+0.03C and MAR-M247 directionally solidified at 1 and 4 $\mathrm{mm}\cdot \min^{-1}$ can be observed, respectively. This intensification of carbide fraction is in line with the increment of carbon content in the superalloys, since carbon is the main carbide forming element (FE). However, the amount of carbides in W4 samples is noticeably lower than in W1 samples. Carbon has been found to alter segregation and alloying elements during solidification [33]. At high cooling rates, solute diffusion is restricted such that carbide FEs enrichment in the interdendritic region occurs to a lesser degree. Accordingly, more carbides are formed at lower cooling rate, which later on leads to a decrease in permeability of the interdendritic area, hindering the flow of the remaining melt.

#### 3.3.2. Morphology

**Figure 5:**
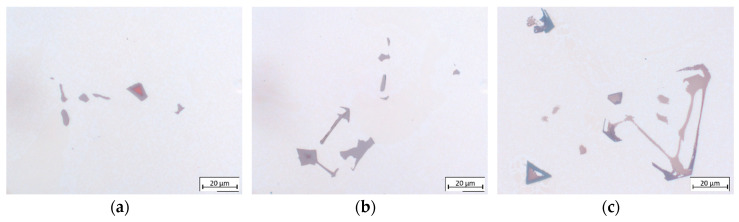
Morphology of carbides in TC-SC samples of: (**a**) CMSX-6; (**b**) CMSX-6+0.03C; (**c**) MAR-M247 directionally solidified at 0.13 $K\cdot s^{-1}$.

**Figure 6:**
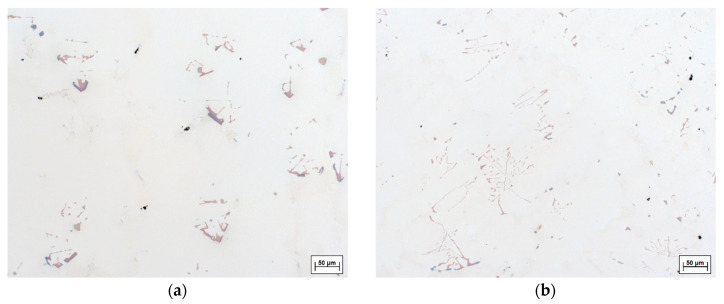
Morphology of carbides in LC-SC samples of MAR-M247 directionally solidified at: (**a**) 0.13 $K\cdot s^{-1}$; (**b**) 0.4 $K\cdot s^{-1}$.

Figure 5 exemplifies the typical carbide morphologies in different superalloys with varying carbon content solidified at the same rate, whereas Figure 6 exhibits differences in carbide morphology across different cooling rates. Carbide morphology is governed by solidification parameters, carbide growth rate, carbide Fes enrichment and surrounding solid geometry, which determines carbide growth space [28,34]. It changes from acicular, nodular and blocky to Chinese script-type with increasing carbon addition and/or cooling rate [35,36]. When carbon content is low, as in CMSX-6 (Figure 5a), the carbide volume is small and the blocky shape provides the smallest surface area [28]. Increasing carbon content, as in CMSX-6+0.03C (Figure 5b), leads to carbides with developing arms from the octahedron apex. Further increment of carbon addition, observed in Figure 5c, results in formation of carbides in different shapes ranging from the emergence of even more arms developed in many directions, forming holes-containing or arrow-shaped carbides to fine needle-like or Chinese script-type carbides.

As explained by Li et al., higher carbon content leads to a greater supersaturation in the later stage of solidification, which induces the growth of well-developed secondary and tertiary dendrite arms [35]. Even though script-like carbides have a large surface area, some orientation relationships between two phases provide lower unit interface and strain energies. Therefore, a solidification system with a large volume of carbides has the lowest free energy when the shape of the carbides is script-like [28]. Figure 6 illustrates that carbide size and shape are significantly influenced by the cooling rate. At low cooling rates, carbide forming elements such as C, Ti, Ta and Hf accumulate at the bottom of the mushy zone which solidifies very slowly. Therefore, the carbides formed are large and have faceted equilibrium morphology. As the cooling rate increases, the decreasing diffusion time hinders carbide growth, resulting in a finer carbide structure [37].

#### 3.3.3. Chemical Composition

**Figure 7:**
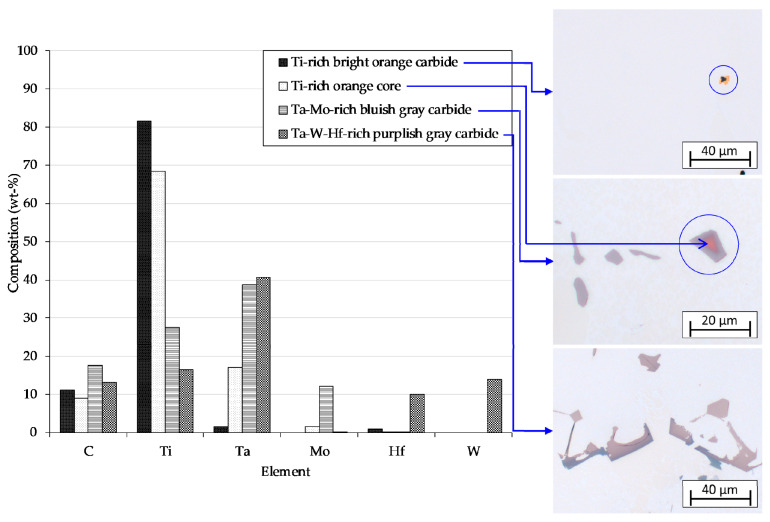
Chemical composition of various carbides observed in OM images of CMSX-6 (W4, **upper**), CMSX-6 (W1, **middle**) and MAR-M247 (W1, **lower**).

According to EDX analysis results, carbides found in this study fall primarily under the MC-type with M dominated by Ti and Ta. Small alumina particles were observed to exist at the cores of a small portion of the faceted carbides, which were reported as well by Fernandez et al. and Zhao et al. [19,32]. These alumina particles are believed to originate from the shell mold material that fell out during investment casting in the Bridgman furnace. Since the melting point of alumina particles used was approximately 2323 K, they remained solid throughout the experiment. As undercooling prevailed during solidification, their surfaces automatically became a preferred nucleation site for the carbides. Direct growth from the melt requires more energy, whilst heterogeneous nucleation on alumina particles reduces the initial necessary energy since surfaces for nucleation are provided. Figure 7 shows the chemical composition of various carbides observed under OM. The first carbides to nucleate on the alumina particle surface are orangish in color, less in Al and O, but more in Ti, Ta, Mo and C than the core. The particle is further encircled by a bluish gray carbide layer consists of more Ti, Ta, Mo and C, but less Al and O than the carbide within.

Another portion of carbides without inclusion of alumina particles is also built up by two distinct colors having two different chemical compositions, i.e., an orangish core and a bluish gray overlay, while the rest of the carbides are completely bluish gray in color. On the other hand, the alloy with extremely low amount of carbon contains carbides in bright orange, which indicates that the carbides are rich in Ti whose content may reach approximately 81 wt.%. The bluish gray color represents carbides with high amount of Ta (~39 wt.%) and Mo (~12 wt.%) but not as much Ti (~27 wt.%). In MAR-M247 samples, carbides with another distinct composition, i.e., rich in Ta (~40 wt.%), W (~14 wt.%) and Hf (~10 wt.%) but less in Ti (~16 wt.%) and Mo (nearly zero), were found. The content of carbon in each carbide varies as well, from ~9–11 wt.% in bright orange and orangish carbides to ~18 wt.% in bluish gray carbides. Since carbide nucleation and growth are controlled by the cooling rate and carbide FEs enrichment during *γ* dendrite growth, this variation in chemical composition implicates that carbides are formed through several stages [20,34].

The bright orange carbide is the first carbide to form at low carbide FEs, particularly at limited amounts of carbon as in CMSX-6, and high cooling rate, at which solute diffusion decreases. Accordingly, the carbides formed are exceptionally rich in Ti as the main constituent besides carbon. At lower cooling rate (CMSX-6 (W1)) and/or higher carbon level (CMSX-6+0.03C (W1 and W4)), either more time for solute diffusion or more carbide Fes or even both are available. At the beginning of solidification when elemental segregation is not yet significant, orangish carbides rich in Ti and some Ta are formed. As solidification proceeds, elemental segregation and thus carbide Fes enrichment in the interdendritic region become more pronounced. Correspondingly, the formed orangish carbides grow in the melt with greater amount of carbide Fes such that a different carbide overlay is produced. At the lower part of the mushy zone where more carbides preferentially nucleate, an adequate amount of carbide FEs has substantially segregated into the interdendritic area so that carbides grown therein are entirely bluish gray in color with richer Ta, Mo and C contents. 

In MAR-M247 samples, another different appearance of carbides was observed that is mainly caused by the chemical composition of the alloy itself. The MAR-M247 alloy contains 10 wt.% of W and a greater amount of Hf and Ta but is less in Ti and Mo. Consequently, the carbides formed are purplish gray in color, loaded with Ta, W and Hf. Some faceted carbides with growing arms in MAR-M247 (W4) sample were found to have a completely different composition, i.e., depleted in Ti and Ta but rich in W (~18–20 wt.%) and Cr (~21–22 wt.%). Previous studies reported that the MC-carbides in MAR-M247 may decompose into M_6_C-and/or M_23_C_6_-carbides during prolonged heat treatment or exposure [21,38]. However, the chemical composition of these carbides is nowhere near that of M_6_C-or M_23_C_6_-type carbides found in prior investigations [38,39]. It is possible that these carbides are the frozen form of the early stage of MC-carbide decomposition.

#### 3.3.4. Crystallographic Orientation

**Figure 8:**
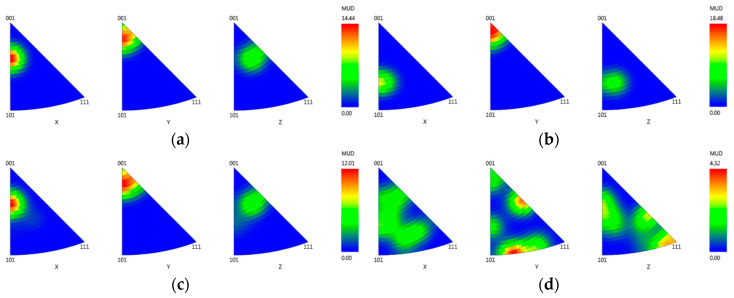

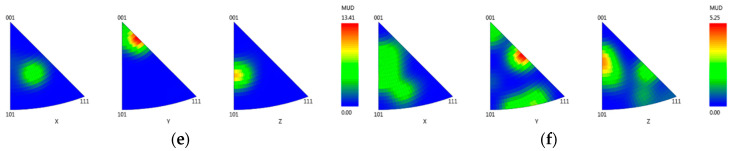
Inverse pole figures of: (**a**,**b**) Ni; (**c**,**d**) TaC; (**e**,**f**) TiC in LC-SC samples of CMSX-6+0.03C directionally solidified at 0.13 $K\cdot s^{-1}$ (**a**,**c**,**e**) and 0.4 $K\cdot s^{-1}$ (**b**,**d**,**f**).

Since carbides are repeatedly reported to grow directly from the melt, they become one of the structure-building phases emphasized in the crystallographic analysis. Accordingly, inverse pole figures (IPFs) of TaC and TiC as the dominant carbide constituent are constructed along with that of Ni as the matrix as depicted in Figure 8. A greater tendency for misorientation can be clearly observed at higher cooling rate. The strength of clustering of poles in Ni IPF of CMSX-6+0.03C (W4) shifts further from 〈001〉 and closer to 〈101〉, while the pole clustering strength in IPF of both carbides are unevenly scattered. At lower cooling rate, the pole clustering strength in all IPFs generally congregates at 〈001〉. This might be caused by the narrow and deficient time provided for atoms in the carbides to collocate at high cooling rates.

### 3.4. Formation and Crystallographic Analysis of *γ*/*γ*’ Eutectic

**Figure 9:**
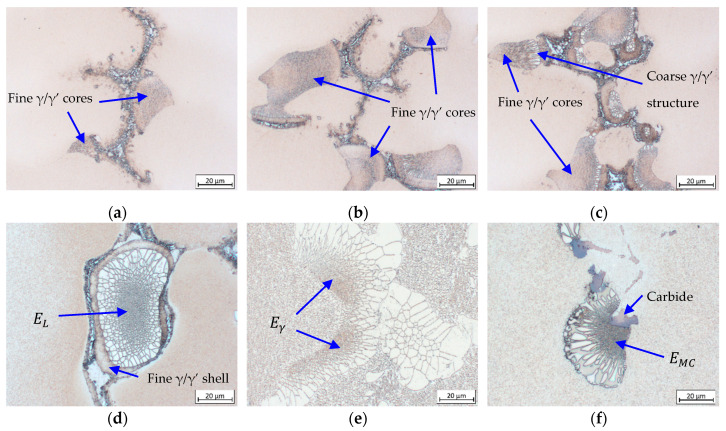
*γ*/*γ*’ eutectic formation: (**a**–**c**) formation sequence observed in LC-MZ sample of CMSX-6 (W4) and (**d**–**f**) the three distinct types of *γ*/*γ*’ eutectic islands found in all samples ((**d**) E_L_; (**e**) E_*γ*_; (**f**) E_MC_).

Figure 9 shows the early stages of *γ*/*γ*’ eutectic formation in the LC-MZ sample of CMSX-6 (W4), which were also observed in other alloys. The *γ*/*γ*’ growth direction begins with the fine *γ*/*γ*’ structure on the *γ* dendrite lobe as shown in Figure 9a. These fine cores growing in size (Figure 9b) were found prior to the coarse *γ*/*γ*’ structure (Figure 9c), which was also discovered in other investigations [14,16,40]. This indicates that the fine *γ*/*γ*’ cores are crystalized by means of a eutectic reaction on different sites, followed by a progressive transition to growth of the coarse *γ*/*γ*’ structure. The eutectic islands then spread three-dimensionally, into the residual melt and the *γ* dendrite, in different directions until they come into contact with each other or a neighboring primary *γ* dendrite. This finding is further supported by calculations of the liquidus temperature of the fine *γ*/*γ*’ cores which is higher than that of the coarse structure [14].

Fine *γ*/*γ*’ shells ahead of the coarse *γ*/*γ*’ structures illustrated in Figure 9d are believed to form during the quenching process instead of the solidification process since it is no longer present in the completely solidified single crystal section [40]. Three distinct types of eutectic islands as depicted in Figure 9d–f were observed throughout the mushy zone and single crystal sections of all samples. They are classified according to the position of their fine *γ*/*γ*’ cores, i.e., thoroughly enveloped by the coarse structure (E_L_), attached to the surface of *γ* dendrite arms while also being partially enclosed by the coarse structure (E_*γ*_) and attached to a carbide surface (E_MC_). The latter type is highly dependent on the carbon level or carbide fraction in the alloy. Increasing carbon content and hence intensifying carbide fraction lead to an increase in the quantity of these eutectic islands.

**Figure 10:**
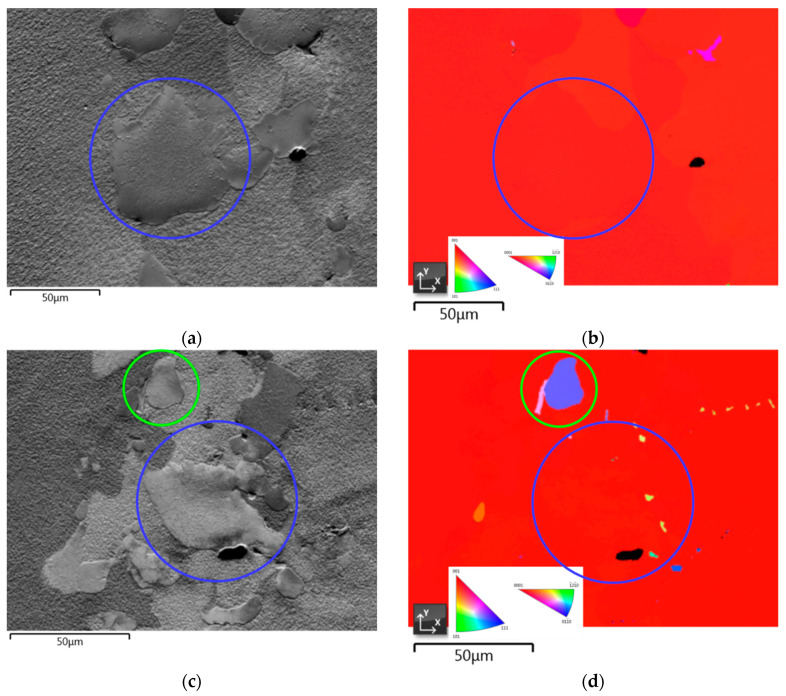
EBSD analysis results of LC-SC samples of CMSX-6+0.03C showing: (**a**,**c**) SEM images; (**b**,**d**) IPF mapping directionally solidified at 0.13 $K\cdot s^{-1}$ (**a**,**b**) and 0.4 $K\cdot s^{-1}$ (**c**,**d**). The blue circles represent the E_*γ*_-type eutectic islands, while the green circle refers to the E_MC_ type.

EBSD analysis performed was focused on the E_L_ and E_MC_ types to determine the possibility of homogeneous nucleation of *γ*/*γ*’ eutectic and the extent of misorientation caused by carbides, respectively. Moreover, the E_*γ*_ type is believed to nucleate on the surface of the *γ* dendrite since its fine core formed in the early stages is generally attached to the dendrite and, hence, their crystallographic orientation is similar. The crystallographic analysis results of LC-SC samples of CMSX-6+0.03C directionally solidified at different rates are shown in Figure 10. There is not any evidence of homogeneous nucleation of the *γ*/*γ*’ eutectic, which further signifies that the E_L_ and E_*γ*_ types are in fact the same. As suggested by Heckl et al., since neither misorientations nor grain boundaries were present, nucleation and growth of the eutectic occur epitaxially on the primary solidified *γ* dendrite [14]. Accordingly, the eutectic adopts the crystal structure of the parent phase, *γ* dendrite. Various 2-D structures might be produced by the same parent *γ*/*γ*’ structure depending on the sectioning orientation and distance from the fine eutectic core [14].

On the other hand, the EBSD analysis result on the E_MC_-type eutectic discloses the expanding influence of misorientation of the carbides as seen in Figure 10c,d. This small eutectic island seems to adopt the crystallographic orientation of the corresponding carbide as their IPF mapping colors are quite similar. Thus, it confirms that the *γ*/*γ*’ eutectic nucleates on the surface of MC-carbide as well. As explained by Wang et al., even though there is a greater tendency for *γ*/*γ*’ eutectic to nucleate on *γ* dendrites than MC-carbides due to their complete coherent crystal relationship, nucleation of *γ*/*γ*’ eutectic on MC-carbides can also be promoted by elemental segregation [41]. Carbide FEs are drawn from the residual liquid to form MC-carbides, while other elements such as Al, Co, Cr and Ni are rejected and enriched encompassing the crystallized carbides. This creates an excellent condition for *γ*/*γ*’ eutectic to nucleate since Ni, Co and Cr are the *γ* FEs, whereas Al and Ni are *γ*’ FEs. The FEs enrichment ahead of MC-carbides merely exceeds the saturation level, which results in a substantial undercooling that eases the *γ*/*γ*’ eutectic nucleation. Upon growing, they exchange their respective FEs such that a co-growing mode occurs.

A prior investigation reported that some E_L_-type eutectic islands were found to have a different orientation from the *γ* matrix [25]. These eutectic islands were suggested to nucleate homogeneously. However, direct growth of eutectic islands from the melt is considered to be highly unlikely since heterogeneous nucleation rate is much larger than homogeneous nucleation rate, especially at small undercooling [42]. Aside from the necessity of sufficiently high undercooling, homogeneous nucleation occurs in the absence of intermediary surfaces for heterogeneous nucleation, while the *γ*/*γ*’ eutectic nucleation takes place in areas bounded by intermediary surfaces of *γ* dendrites and MC-carbides. A more reasonable explanation would be that the eutectic islands nucleated on the surfaces of MC-carbides, instead of growing from the melt, but the corresponding carbides were not visible. This could be caused again by the dependence of 2-D structure on the sectioning position and orientation of the whole 3-D structure. Therefore, a further and detailed 3-D analysis on the *γ*/*γ*’ eutectic islands is necessary to gain a more assertive assessment of their nucleation mode.

### 3.5. Volume Fraction and Morphology of *γ*’ Precipitates

**Figure 11:**
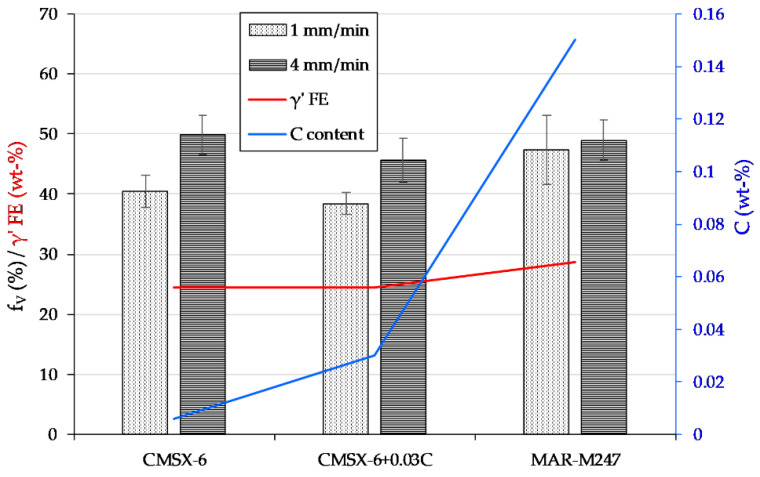
Volume fraction of *γ*’ precipitates in the single crystal part of the samples directionally solidified at different rates.

Assuming that the *γ*’ phase precipitated in ideal-cubic morphology with a completely identical size whose particle distribution was uniform such that the channel width was the same everywhere, *γ*’ volume fraction $f_{v}$ was calculated from the area fraction [43]. The *γ*’ area fraction was obtained with the assistance of ImageJ. The calculation results are plotted in Figure 11 in juxtaposition with the compositions of carbon and *γ*’ FEs. The latter includes Al, Ti and Ta, which are beneficial for inducing *γ*’ precipitation, as well as Cr, Mo and W, which are functional for enhancing the *γ*’ volume fraction [10]. A strong correlation of withdrawal and thus cooling rate on the $f_{v}$ of the *γ*’ precipitates could be observed. In that regard the *γ*’ particles at higher cooling rate are finer and denser, which is supported by calculation results signifying greater values of $f_{v}$ and a previous study [44]. The maximum and minimum values of $f_{v}$ of 49.8% and 38.39% are established in CMSX-6 and CMSX-6+0.03C solidified at 0.4 $K\cdot s^{-1}$ and 0.13 $K\cdot s^{-1}$, respectively. 

Higher cooling rate results in greater undercooling and therefore a finer and denser structure of *γ*’ precipitates, as the maximum solid-solid transition rate is seen at larger undercoolings due to deceleration of diffusion with decreasing temperature [45]. However, the $f_{v}$ is not merely influenced by the cooling rate, but also the *γ*’ FEs and carbon content. The *γ*’ FEs affect the overall tendency of $f_{v}$ of the alloys compared with one another, which is similar between CMSX-6 and CMSX-6+0.03C while it is rather higher for MAR-M247. The effect of *γ*’ FEs is, however, slightly toned down by the presence of carbon. Carbon bonds Ti, Ta, Mo and W to form carbides in the interdendritic region just below the outset of solidification. The content of these elements in the interdendritic area is depleted to a certain extent throughout the development of *γ* dendrites alongside the formation of carbides before the *γ*’ phase is precipitated. Concurrently, *γ* dendrites grow in the expense of depleted melt in the interdendritic region, which causes the dendrites to contain lesser *γ*’ FEs. This condition ultimately leads to a decrease in $f_{v}$ of alloys with additional carbon.

## 4. Conclusions

The microstructure evolution and solidification sequence for several Ni-based superalloys has been investigated by directional solidification experiments combined with a quenching technique. Aside from that, the influence of carbon and alloying elements and the effect of the withdrawal respective to the cooling rate has been studied. Another focus was directed to the evaluation of the relationship between carbide precipitation and the formation of other phases like the *γ* matrix and *γ*/*γ*’ eutectic.

The following conclusions can be drawn:

- The solidification sequence of CMSX-6, CMSX-6+0.03C and MAR-M247 begins with the crystallization of *γ*, which can be described by $L_{1}\to L_{2}+\gamma$ at 1476.88–1521.15 K. Besides the primary γ dendrites, some equiaxed carbides are observed in the interdendritic region, indicating the direct growth of carbides from the melt through the reaction $L_{2}\to L_{3}+\gamma +MC$ at 1442.58–1496.56 K. Subsequently, the formation of the *γ*/*γ*’ eutectic proceeds via both eutectic $L_{3}\to \gamma /\gamma^{\prime }$ and peritectic $L_{3}\;+\;\gamma \to \gamma /\gamma^{\prime }$ reactions at 1418.26–1472.56 K. The last forming phase is *γ*’, which forms by means of solid-state phase transformation reaction from $\gamma \to \gamma^{\prime }$ at 1405.68–1470.29 K.
- An increase in carbon content causes and intensifying porosity for both applied cooling rates. Nevertheless, the porosity amount at the higher cooling rate is rather toned down to a smaller fraction of carbides formed. The presence of carbides in the interdendritic area notably reduces its permeability which hinders the melt flow.
- EDX analysis indicates that the carbides found in this study mainly correspond to the MC-type, whereby M is dominated by Ti and Ta. At higher carbon concentrations and/or lower cooling rates, the first carbides to precipitate are orangish in color and rich in Ti, which are later on enveloped by a bluish gray overlay loaded with Ta and Mo. In MAR-M247 samples, the carbides observed are rich in Ta, W and Hf, having a purplish gray color. The carbide morphology changes from faceted to Chinese script-type for increasing carbon concentration respective to cooling rate. The size and fraction of the carbides decrease with increasing cooling rate, but the carbide fraction is primarily governed by the carbon content of the alloy. A greater tendency for carbide misorientation and, hence, the presence of local polycrystalline structures can be observed at higher cooling rates. Accordingly, the single crystalline character of the alloy is retained better, applying lower cooling rates.
- The growth direction of *γ*/*γ*’ eutectic begins with the fine *γ*/*γ*’ lamellar core as it was found prior to the coarse *γ*/*γ*’ structure. The fine eutectic core is crystallized by means of a eutectic reaction, followed by a progressive transition to growth of the coarse structure. The growth continues until it comes into contact with other eutectic islands or a neighboring primary *γ* dendrite. Crystallographic analysis on CMSX-6+0.03C samples indicates that there is no evidence of homogeneous nucleation of *γ*/*γ*’ eutectic. The eutectic islands nucleate on the surface of *γ* dendrites, and thus the eutectic adopts the crystal structure of the dendrite. A small eutectic island observed to have the same orientation as a carbide but a different orientation from the dendrite confirms that *γ*/*γ*’ eutectic does not only nucleate on the dendrite lobe but also on the carbide surface.
- Increasing cooling rate leads to finer and denser *γ*’ precipitates. The *γ*’ volume fraction is influenced by *γ*’ FEs, but the effect is slightly depleted by the presence of carbon. Carbon bonds *γ*’ FEs to form carbides in the interdendritic area so that the *γ*’ FEs are consumed while *γ* dendrites are growing. The solidified *γ* dendrite just before *γ*’ precipitation becomes more depleted in *γ*’ FEs as carbon level in the alloy increases and thus the *γ*’ volume fraction decreases.

## Figures and Tables

**Table 1:** Chemical composition of the employed Ni-based superalloys in wt.%.

Alloy	Cr	Co	Mo	W	Al	Ti	Ta	Hf	C	B	Ni
CMSX-6	10	5	3	-	4.8	4.7	2	0.1	0.006	-	Bal.
CMSX-6+0.03C	10	5	3	-	4.8	4.7	2	0.1	0.03	-	Bal.
MAR-M247	8.4	10	0.7	10	5.5	1	3	1.5	0.15	0.015	Bal.
